# Cell-Type-Specific Proteomics: A Neuroscience Perspective

**DOI:** 10.3390/proteomes6040051

**Published:** 2018-12-09

**Authors:** Rashaun S. Wilson, Angus C. Nairn

**Affiliations:** 1 Yale/NIDA Neuroproteomics Center, 300 George St., New Haven, CT 06511, USA; rashaun.wilson@yale.edu; 2 Department of Psychiatry, Yale School of Medicine, Connecticut Mental Health Center, New Haven, CT 06511, USA

**Keywords:** cell type, neuroscience, proteomics, mass spectrometry, neuron, proximity labeling, affinity chromatography, neuroproteomics, biotinylation

## Abstract

Cell-type-specific analysis has become a major focus for many investigators in the field of neuroscience, particularly because of the large number of different cell populations found in brain tissue that play roles in a variety of developmental and behavioral disorders. However, isolation of these specific cell types can be challenging due to their nonuniformity and complex projections to different brain regions. Moreover, many analytical techniques used for protein detection and quantitation remain insensitive to the low amounts of protein extracted from specific cell populations. Despite these challenges, methods to improve proteomic yield and increase resolution continue to develop at a rapid rate. In this review, we highlight the importance of cell-type-specific proteomics in neuroscience and the technical difficulties associated. Furthermore, current progress and technological advancements in cell-type-specific proteomics research are discussed with an emphasis in neuroscience.

## 1. Introduction

Novel methods for proteomic analysis of biological tissues have developed rapidly in the past decade; however, neuroproteomics remains a challenging field of study. The mammalian central nervous system (CNS) is far different from any other organ in the mammalian system, primarily because it is made up of several hundred different cell types [1]. Each cell type has unique characteristics, and distinct populations of cells are present in different brain regions. For instance, although 40% of all cells in the brain are astrocytes, neurons outnumber astrocytes in the cerebellum, whereas there is an inverse correlation in the cortex [2]. Furthermore, Herculano-Houzel et al. [3] determined that almost 70% of the two billion neurons found in the adult rat brain are located in the cerebellum, and five-fold less are present in the cortex. Brain cells also possess region-specific identities and biomarkers that have proven useful in cell-type-specific studies but can also complicate analyses [4,5]. In addition, neural cells lack uniformity and make projections to different brain regions, resulting in spatiotemporal regulation of many signaling processes within the brain. Consequently, these factors make separation and isolation of specific cell types from brain challenging.

A second issue is that proteomic analysis of brain cells has lagged behind in comparison to its transcriptomic counterpart, which continues to make rapid advances. The facile method of RNA amplification has enabled over 500 single-cell transcript expression analyses [6]. In a few years, the field has moved from the use of quantitative reverse transcription-polymerase chain reaction (qRT-PCR) to quantify globin gene expression in human erythroleukemic cells [7] or measure expression levels of five genes in single cells isolated from mouse pancreatic islets [8], to methods with greater scope and scale. For example, RNA sequencing (RNA-seq) methods have been used to successfully analyze gene expression in single cells [9,10,11,12,13,14]. One study classified 3005 cells in the mouse cortex and hippocampal CA1 region using single-cell RNA-seq, revealing 47 subclasses from nine known cell types [13]. A later report used single-nuclei RNA-seq to identify 16 neuronal subtypes from 3227 single-neuron datasets isolated from six different regions of the postmortem human brain [14]. Recently, a study successfully profiled gene expression in 4347 single cells from mutant human oligodendrogliomas [10]. Variations of the RNA-seq method have been developed to enable more high-throughput, comprehensive analyses [15,16], including a recent study that profiled over 400,000 single-cell transcriptomes from more than 800 mouse cell types using a method termed Microwell-seq [15]. This rapid, cost-effective method uses an agarose microwell system for single-cell isolation and barcoded magnetic beads for mRNA capture. Drop-seq uses a similar concept but isolates and lyses single cells in nanoliter droplets of liquid prior to barcode labeling [16,17,18,19]. This method enabled isolation and characterization of over 44,000 transcriptomes from mouse retinal cells, which were ultimately grouped into 39 different cell types [16]. Drop-seq has also been used to analyze RNA expression levels in 690,000 cells from 9 different adult mouse brain regions [18]. Though comprehensive transcriptomic analyses have proven useful in the characterization of specific cell types, these methods do not account for differential control of protein synthesis and degradation. Therefore, mRNA expression often does not correlate with protein abundance and may not be reliably used as a predictive tool for proteomics [20].

Large-scale proteomic studies use mass spectrometry, an approach that continues to improve in terms of accuracy and sensitivity [21,22,23,24]. However, one major difference between transcriptomic and proteomic profiling is that protein abundance cannot be amplified in the same way that nucleic acids can. Therefore, the protein quantity isolated from a cell population must be above the threshold of detection for mass spectrometry analysis. While highly abundant proteins can be analyzed by mass spectrometry at the single cell level (see below), the protein yields obtained from a single cell are often below the levels necessary for reliable quantitation and therefore do not allow the depth of coverage observed in transcriptomic analyses. Moreover, past and current cell isolation techniques are often inefficient and collect small quantities of cells in a given experiment, which in turn results in low protein yields. Specific to neurons and other CNS cells, due to their non-uniformity of size and subcellular organization, many of the current separation techniques are incapable of retaining cellular structure, often resulting in leakage of cellular contents or loss of cell integrity entirely. Furthermore, protein/peptide loss can occur during sample preparation, either through peptide adsorption to sample tubes and/or during transfer of sample to and from multiple tubes [25,26]. Mass spectrometry analysis itself can also influence the number of proteins identified, which can often be attributed to ionization efficiency and instrument sensitivity [26].

Overcoming the challenges facing cell-type-specific proteomics is of critical importance, as many types of psychiatric, developmental, and neurodegenerative disorders are associated with specific cell types in the brain. Drug addiction is one of these psychiatric disorders in which specific neuronal cell types are implicated. For instance, the psychostimulant, cocaine, regulates the reuptake of the neurotransmitter, dopamine, leading to aberrant signaling in specific sub-types of striatal medium spiny neuron (MSN) in the dorsal and ventral striatum [27]. While morphologically similar, MSNs can be separated into at least two large subtypes that differentially express D1- or D2-classes of dopamine receptors that are in turn differentially coupled to either increased or decreased cAMP signaling, respectively [28,29]. Thus, exposure to cocaine results in opposite patterns of phosphorylation of important intracellular targets such as DARPP-32 in intermixed sub-populations of MSNs [29]. Biochemical analysis of striatum, in the absence of separation of different MSN cell types, leads to an averaging of the increased or decreased signals, and a loss of important information.

In addition to drug addiction, neurodegenerative disorders like Alzheimer’s disease (AD) and Down syndrome (DS) are associated with specific cell types in the brain [30,31]. For instance, pathology of both AD and DS patients involves overproduction of amyloid beta peptide, and the development of neurofibrillary tangles and amyloid plaques. Astrocytes, which are a type of glial brain cell, also play active roles in pathogenesis of AD brain tissue [5,32]. In mice overexpressing amyloid beta, plaques are surrounded by reactive astrocytes and activated microglia [33,34]. Furthermore, brain inflammation caused by glial and microglial activation is observed in brain tissue of AD patients [33,35,36].

Other cell-type-associated disorders include Parkinson’s disease (PD), Amyotrophic lateral sclerosis (ALS), and Huntington’s disease (HD). In PD subjects, pathology within the *substantia nigra* revealed a loss of a sub-population of dopaminergic neurons, followed by an increase in Lewy body structures within the retained neurons [5,37,38]. The subsequent DA depletion causes cell-specific effects such as hyper- and hypoactivation of D2 and D1 MSNs, respectively [39,40,41]. Astrocytes are also implicated in PD in many animal-based studies [5]. ALS is a degenerative disease that affects the motor cortex, brain stem, and spinal cord and ultimately results in motor neuron death [5,42,43]. Patients with HD exhibit a preferential loss of D2 MSNs, and an accumulation of the mutant form of Huntingtin (HTT) protein occurs in human neurons and astrocytes [5,44,45].

It is clear from the ongoing list of disorders that a greater focus needs to be placed on biochemical characterization of neural cell types. Though many technologies have advanced in recent years to address the issues of cell separation and isolation as well as increasing the depth of proteomic coverage for cell-type-specific analyses, there are still many aspects that need to be improved. This review will outline the different methods available, while also noting the benefits and limitations of each. Studies which have employed these techniques will also be highlighted, and potential improvements for these methods will be discussed.

## 2. Cell-Type-Specific Isolation Methods

The nonuniformity and complex networks of different cell populations within the brain often require the use of cell-type-specific markers to improve the accuracy of isolation. This can be accomplished through promoter-directed expression of a reporter protein either through viral transduction (transient) or generation of a transgenic animal (stable). While viral transduction can be useful for some experimental applications (See Proteome labeling methods), expression levels may be variable when compared to transgenic animals, which may ultimately affect proteomic analyses. Though generation of transgenic animals can be time- and resource-intensive, many groups have now successfully developed transgenic tools for characterization of brain cell types [46,47]. One of these tools was developed by taking advantage of a bacterial artificial chromosome (BAC) to express a green fluorescent protein (GFP) marker in specific neural cell types [46]. The same BAC approach was used to generate Ribo-tagged transgenic mice expressing an enhanced green fluorescence protein (EGFP)-L10a ribosomal protein under the control of cell-type-specific promoters [47]. Along with cell-type-specific visualization, this design has the added advantage of enabling translating ribosome affinity purification (TRAP) to isolate ribosomes from target cell types. Emergence of these tools coupled to cell isolation techniques is useful for proteomic analysis of CNS cell types.

**Figure 1:**
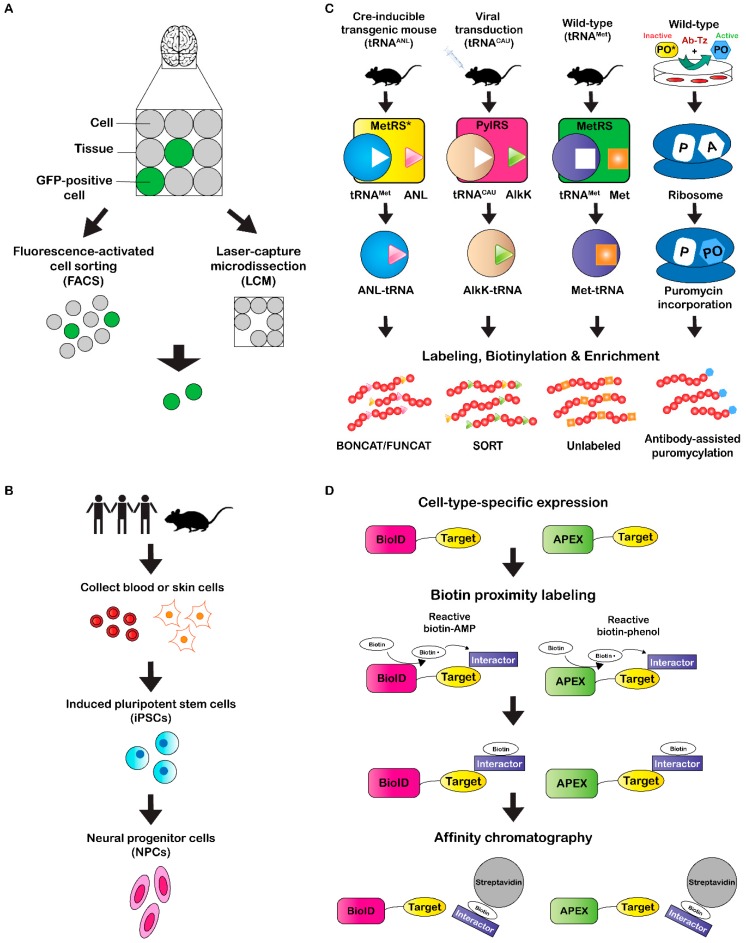
Methods for cell-type-specific isolation and proteome enrichment. (**A**) Two methods for specific cell isolation from a total cell population. Animal models can be generated that express fluorescent markers in a cell type of interest. Fluorescent cells can be detected and isolated using fluorescence-activated cell sorting (FACS) or laser capture microdissection (LCM). FACS requires homogenization of tissue prior to cell sorting, while LCM enables cells isolation from intact tissue slices. (**B**) Basic workflow of induced pluripotent stem cell (iPSC) differentiation. Skin or blood cells are collected from a biological organism of interest and used to generate induced pluripotent stem cells (iPSCs). Factors are then added to iPSCs for differentiation into neural progenitor cells (NPCs). (**C**) Cell-type-specific labeling methods enable stochastic incorporation of a non-canonical amino acid or puromycin into the target proteome. The cell-type-specific expression of a tRNA synthetase is accomplished either by genetic engineering of a Cre-dependent transgenic mouse (BONCAT/FUNCAT) or via viral transduction (SORT). The incorporated amino acid can be further biotinylated for enrichment prior to LC-MS/MS analysis (BONCAT/SORT) or modified with a fluorescent probe for visualization (FUNCAT). Puromycin labeling occurs through introduction of a cell-type-specific enzyme-tagged antibody (Ab-Tz) followed by an inactive puromycin analog. Activation of puromycin occurs after Tz reacts with the inactive puromycin analog. (**D**) Experimental workflow for BioID and APEX proximity labeling techniques. BioID or APEX fusion target proteins are expressed in a specific cell type. Reactive biotin is supplemented, and target interacting proteins are biotinylated via BioID or APEX. Biotinylated interactors can be enriched using affinity chromatography techniques with a stationary phase such as streptavidin prior to LC-MS/MS.

One frequently-used method to isolate specific cell types is fluorescence-activated cell sorting (FACS) (Figure 1A), which relies on a fluorescent cellular marker that can be endogenously-expressed or immunolabeled for detection. In an early study, 5000–10,000 striatal MSNs were isolated via FACS from fluorescently-labeled neurons expressing EGFP under the *Drd1*, *Drd2*, or *Chrm4* promoter (BAC transgenic mice) [48]. FACS of tissue from transgenic mice expressing GFP under the control of the parvalbumin-expressing interneuron (*Pvalb*) promoter was later used to isolate approximately 5000 and 10,000 GFP-positive nuclei from striatal and hippocampal tissue, respectively [49]. Nuclei from different sub-populations of MSNs were also subjected to FACS after acute or chronic cocaine treatment to observe cell-type-specific differential post-translational modification of histones [50]. FACS has also been used for glutamatergic synaptosomal enrichment by expressing fluorescent VGLUT1 protein in mice, which resulted in identification of 163 enriched proteins after mass spectrometry analysis [51]. Recently, FACS and subsequent LC-MS/MS was performed on sensory inner ear hair cells, enabling identification of 6333 proteins [52].

An alternative single-cell isolation method is termed laser capture microdissection (LCM) (Figure 1A), which uses a microscope equipped with a high-precision laser to dissect small areas within a tissue slice (>100 µm^2^). Imaging and dissection can be performed in fluorescence or bright-field modes, enabling a variety of experimental applications. For instance, Drummund et al. [53] performed LCM on neurons isolated from formalin-fixed, paraffin-embedded (FFPE) AD cortical brain tissue, which yielded more than 400 proteins identified by LC-MS/MS analysis. In this study, extensive sample treatment optimization was also performed on tissue isolated via LCM from the temporal cortex. Results from this optimization ranged from 202 to over 1700 proteins identified from approximately 4000–80,000 neurons. Another study identified 1000 proteins from tissue sections of neuromelanin granules isolated from the human *substantia nigra* [54]. Furthermore, mass spectrometry analysis of four different compartments in FFPE fetal human brain tissue identified a total of 3041 proteins [55]. Two recent reports isolated cells from human post-mortem tissue using LCM to identify a small number of potential biomarkers from AD [56] and ischemic stroke [57] patients via mass spectrometry. LCM was also recently used to quantify approximately 1000 proteins from 10–18 cells (100-µm-diameter) isolated from different rat brain regions [26]. For these analyses, optimization was first performed with 50 µm (2–6 cells), 100 µm (10–18 cells), and 200 µm (30–50 cells) diameter tissue sections from rat brain cortex, where 180, 695, and 1827 protein groups were identified, respectively.

While LCM clearly offers precision for a variety of experimental workflows, it does have limitations. If an endogenously-expressed fluorescent protein is used as a cell-type-specific marker in the tissue of interest, it must be expressed at an intensity above the threshold of detection for the microscope to accurately dissect. Furthermore, most LCM microscopes are not capable of cooling the tissue specimen during dissection. Therefore, the user must work rapidly to prevent altered protein expression and/or degradation, particularly when using fresh tissue. Moreover, dissection of the tissue can be more tedious and time-consuming than many other isolation methods, which could result in a lower number of cells (and protein) isolated in a given amount of time. Finally, if the tissue must be immunolabeled, the antibody is often processed with the rest of the cellular protein extract. This could ultimately affect proteomic results depending on the amount of antibody used. Despite these potential issues, LCM is clearly a powerful method that can be useful for many types of cell-type-specific applications.

Although animal models are useful for investigative research in neuroscience, results and treatments do not always translate to the human system. It is difficult to obtain brain tissue from human subjects, particularly over a range of development with age-matched controls and within a post-mortem interval short enough to avoid protein degradation and variations in post-translational modifications (PTMs) [58,59,60,61]. In an effort to address these challenges, researchers have turned to developing specific neuron cell types from induced pluripotent stem cells (iPSCs) (Figure 1B) [62,63]. A major benefit of using iPSCs is that they can be produced from human somatic cells such as dermal fibroblasts (HDF) instead of embryonic stem cells, which have ethical conflicts associated. Furthermore, these iPSCs can be directly reprogrammed to differentiate into virtually any cell type with patient- or disease-specificity [62]. Many studies have already demonstrated successful production of a variety of region-specific neuronal cell types including ventral forebrain cholinergic, ventral midbrain dopaminergic, cortical glutamatergic, and cholinergic motor neurons [64,65,66,67,68]. Recently, iPSCs have undergone proteomic characterization for numerous experimental applications [69,70,71,72,73,74]. For instance, Yamana et al. [69] compared lysates of iPSCs and fibroblast cells to identify a total of 9510 proteins via mass spectrometry analysis. A later study used quantitative mass spectrometry to identify 2217 total proteins in spinal muscular atrophy (SMA) patient-derived and healthy control motor neurons differentiated from iPSCs [73]. A comparison of the two groups indicated that 63 and 30 proteins were up-regulated in control and SMA motor neurons, respectively. Recently, three-dimensional neuron-spheroids were derived from AD and control patient iPSCs and subjected to tandem mass tag (TMT) LC-MS/MS analysis [74], which is a quantitative mass spectrometry approach that uses reporter ions generated during MS/MS fragmentation for quantitation [75]. Collectively, 1855 proteins were identified in the 3D neuro-spheroid samples that were differentiated from a total of ten iPSC lines between both the AD and control subjects. Furthermore, 8 proteins were found to be up-regulated in AD subjects, while 13 proteins were down-regulated. Another recent study profiled the proteomes of iPSCs, neural progenitor cells (NPCs), and differentiated neurons in cell culture to identify a total of 2875 proteins among all three groups [55]. Notably, 90, 33, and 126 proteins were unique to iPSCs, NPCs, and neurons, respectively. Although differentiation of iPSCs has demonstrated significant promise for moving closer to a human model system while also improving protein yield, these analyses are still being performed in vitro. It therefore becomes difficult to maintain true neural connectivity, which could ultimately result in altered protein expression compared to what would normally be observed in the human brain. Nevertheless, this approach still has potential for a variety of neurological applications in the future.

## 3. Proteome Labeling Methods

Cell-type-specific proteome labeling is a technique that can be used to circumvent the issue of maintaining cellular integrity during isolation. Until recent years, proteome labeling studies were performed primarily using Stable Isotope Labeling with Amino acids in Cell culture (SILAC) [76,77,78,79,80,81,82,83]. The obvious caveat to SILAC, however, is that experiments must be performed in cell culture. A variation termed Stable Isotope Labeling with Amino acids in Mammals (SILAM) can be used for quantitation of protein expression in vivo, however, labeling times are long (~25 d) and it cannot be performed in a cell-type-specific manner. Recent efforts have attempted to make in vivo labeling methods compatible with cell-type-specific applications. One of the first studies to perform in situ proteome labeling over a short, 2 h time course, was termed BioOrthogonal Non-Canonical Amino acid Tagging (BONCAT) [84]. BONCAT takes advantage of a cell’s protein synthesis machinery and enables incorporation of a noncanonical amino acid into the proteome of interest (Figure 1C). Recently, this method has transitioned to cell-type-specific labeling of proteomes through generation of transgenic mice that express a mutated methionyl-tRNA synthase (MetRS*) with an expanded amino acid binding site that recognizes the noncanonical amino acid ANL [85]. Expression of MetRS* is driven by a cell-specific promoter and enables charging of supplemented ANL onto an endogenous tRNA^Met^, which is then stochastically incorporated into the target cell proteome. After labeling, click-chemistry can be performed to biotinylate ANL residues, followed by enrichment via streptavidin affinity chromatography. Mass spectrometry analysis of ANL-labeled, enriched proteins in hippocampal neurons and Purkinje cells resulted in 2384 and 1687 proteins identified, respectively [85]. Furthermore, a hippocampal proteome analysis of mice exposed to standard (SC) or enriched (EE) housing environments identified 2384 and 2365 proteins, respectively, of which 225 were significantly regulated after statistical comparison. Not only can click-chemistry be used for biotinylation, but fluorescent probes can be added to the ANL residues, which Dietrich et al. [86], termed FlUorescent Non-Canonical Amino acid Tagging (FUNCAT) (Figure 1C). This method can be used for temporal visualization of newly-synthesized proteins, while also enabling post-visualization enrichment by methods such as immunoaffinity chromatography.

A similar technique called Stochastic Orthogonal Recoding of Translation (SORT) has also recently been established to label proteomes in vivo [87,88]. Instead of requiring generation of a transgenic animal, SORT uses targeted, viral-mediated expression of an orthogonal pyrrolysyl-tRNA synthetase-tRNA_xxx_ pair that recognizes and incorporates a non-canonical amino acid AlkK into the target proteome of interest (Figure 1C). Click-chemistry can then be performed in the same way as BONCAT/FUNCAT. Recently, SORT was used to label, biotinylate, and enrich proteins in mouse striatal MSNs prior to mass spectrometry analysis, which resulted in identification of 1780 cell-type specific proteins [89].

While these methods of cell-type-specific proteome labeling seem advantageous for future studies in neuroproteomics, there are still associated challenges and extensive optimization required for each experiment. For BONCAT/FUNCAT, transgenic animals must be generated and characterized, which is not only time-consuming, but costly. Furthermore, the MetRS* expression levels may vary depending on the cell-type-specific promoter used, which could result in low labeling efficiency and ultimately low protein yield for mass spectrometry analysis. Similarly, low expression levels of the pyrrolysyl-tRNA synthetase-tRNA_xxx_ pair could also be observed for the SORT method for a variety of reasons including promoter selection, transduction efficiency, and accuracy of injection. Both methods also require supplementation of the non-canonical amino acid, either through drinking water intake or injection. This supplementation also needs to be optimized to ensure equivalent dosages and labeling efficiencies occur between animals. Moreover, the proteomics results from the aforementioned studies [85,89] indicate that improvements need to be made to reach a greater depth of proteomic coverage. The observed number of protein identifications is far below the known upper limit of detection (~12,000 proteins) [90,91] and could potentially be improved by a variety of factors such as increasing the number of animals used and/or selecting a promoter that labels at a level above the limit of detection for the assay but does not label proteins at a level that could interfere with cellular processes.

Another labeling approach that takes advantage of the cell’s native protein synthesis machinery uses a puromycin analog tag [92,93,94,95]. The puromycin analog binds the acceptor (A) site of the ribosome and is then incorporated into the nascent polypeptide chain prior to inhibition of protein synthesis. The incorporated puromycin analog can then be chemically modified to enrich for newly synthesized proteins. This method was first demonstrated in cultured cells and mice using *O*-propargyl-puromycin (OP-puro), where newly-synthesized proteins were visualized via fluorescence microscopy after a copper(I)-catalyzed azide-alkyne cycloaddition (CuAAC) reaction with a fluorescent azide [92]. Recently, a similar technique was modified for cell-type-specific labeling of proteomes in vivo [94]. This modification involves introduction of a cell-type-specific antibody bearing a tetrazing (Tz) tag and a “caged” form of puromycin (TCO-PO), which is unable to be incorporated into the proteome. When the Tz-tagged antibody and a TCO-PO molecule come in contact, a reaction occurs which results in conjugation of TCO to the antibody, rendering the PO molecule “uncaged” and free to incorporate into the proteome of the target cell. From this study, more than 1200 proteins were identified via LC-MS/MS when this method was employed in A431 cells. An earlier study performed a similar type of experiment with cell-type-specific, viral-mediated expression of an enzyme capable of activating a “caged” puromycin analog in mouse pancreatic islets and HEK 293T cells [95]. Mass spectrometry analysis of the HEK 293T cell proteome resulted in identification of 1165 proteins enriched puromycin-incorporated, enzyme-expressing proteome.

There are several advantages to using a puromycin labeling strategy over the biorthogonal labeling methods. First, the functional concentration of puromycin is much lower than that of noncanonical amino acids, reducing the likelihood of unwanted side-effects [92,94,95,96]. Furthermore, unlike noncanonical amino acids, methionine does not directly compete with puromycin for incorporation into the proteome. Therefore, animals that undergo puromycin labeling do not require the low-methionine diet which may be necessary for biorthogonal labeling methods and are not subject to potential bias toward proteins with higher methionine content [92,93]. Another advantage is that puromycin incorporation may not require use of a genetically modified organism, which does not always represent a true native biological environment [94]. Moreover, puromycin incorporation displays higher temporal resolution than biorthogonal labeling, which requires charging of the non-canonical amino acid to the tRNA prior to incorporation [92,93,94]. Despite the advantages of in vivo puromycin incorporation, cell-type-specific variations have only been demonstrated in cultured cells to date [94,95].

Not only are specific cellular proteomes being labeling for general protein identification, but in situ proximity labeling methods have recently emerged to identify protein-protein interactors within discrete cellular compartments. In general, these methods rely on expression of a promiscuous biotin protein ligase fused to a target protein whose interacting proteins are being investigated. After biotin supplementation, the target interacting proteins are biotinylated by the ligase and can then be enriched and identified using proteomic analysis (Figure 1D). One of these methods has been termed BioID, which was originally developed by Roux et al. [97] and used to identify lamin-A (LaA) interacting proteins. In this study, an *E. coli* biotin protein ligase BirA was fused to LaA and expressed in HEK293 cells to identify 122 proteins unique to BioID-LaA via LC-MS/MS. A more recent study used the BioID method to identify interacting proteins of excitatory and inhibitory postsynaptic protein complexes [98]. Viral-mediated expression of BirA, PSD-95-BirA, or BirA-gephyrin, BirA-collybistin, and BirA-InSyn1 was performed in mouse brain tissue prior to enrichment of biotinylated proteins and subsequent mass spectrometry analysis. For the PSD analysis, PSD-95-BirA interacting proteins were compared to those of the BirA control. In total, 2183 proteins were identified, 121 of which were enriched at least two-fold in PSD-95-BirA samples compared to the BirA control. For the inhibitory protein complexes, gephyrin-, collybistin-, and InSyn1-BirA interacting proteins were compared to those of the BirA control. Mass spectrometry analysis of the samples identified 2533 total proteins with a combined 181 proteins significantly enriched in the three target interactomes compared to the BirA control. More recently, BioID2 was developed, which is a similar method that employs a smaller promiscuous biotin ligase [99]. This improved method has several advantages to traditional BioID, including increased selectivity of targeting fusion proteins, a reduced amount of biotin required, and enhanced labeling of proximal proteins. TurboID is a similar approach developed recently that takes advantage of a different mutated form of biotin ligase, which is capable of proximity labeling within 10 min [100]. In this study, TurboID displayed a significantly higher biotin labeling efficiency and a similar proteome coverage of subcellular compartments within HEK293T cells after quantitative LC-MS/MS when compared to BioID.

A second method termed APEX (short for Enhanced APX) uses an engineered ascorbate peroxidase fusion protein for biotin labeling of target interacting proteins. This method was first demonstrated in HEK293 cells, where APEX was targeted to the mitochondrial matrix, and biotinylated interacting proteins were enriched and subjected to LC-MS/MS [101]. In total, 495 proteins were identified in the mitochondrial matrix proteome. Recently, APEX was used in *C. elegans* to identify tissue-specific and subcellular-localized proteomes [102]. APEX was targeted to the nucleus or cytoplasm of intestine, epidermis, body wall muscle, or pharyngeal muscle tissues, from which 3180 interacting proteins were collectively identified. A separate study used APEX to identify spatiotemporal interacting proteins of the delta opioid receptor (DOR) in HEK cells [103]. This study observed changes in DOR interactions over an activation time course of 1–30 min as well as different subcellular compartments, including the plasma membrane (PM) and endosome (Endo). Recently, a modified APEX strategy was used to map proteins at excitatory and inhibitory synaptic clefts of rat cortical neurons, resulting in identification of 199 and 42 proteins, respectively [104].

Like the other labeling techniques, extensive optimization of these proximity labeling assays is required for optimal performance. Moreover, the amount of starting material needed for adequate protein enrichment for LC-MS/MS analysis is substantial and not feasible for small amounts of tissue or certain cell types. Furthermore, standardization and reproducibility of labeling methods becomes difficult since protein output is often not provided (See Table A1) and can vary between organisms. Though these proximity labeling methods are similar in practice, APEX labeling times are much faster (~1 min) compared to the 24 h labeling time of the BioID method, which could significantly impact proteomics results. Notably, however, APEX has limited stability in heated or reducing environments compared to BioID, and the presence of H_2_O_2_ in the cell can lead to toxicity. Nevertheless, APEX does have great appeal, particularly for those interested in rapid proteomic changes such as altered subcellular localization or metabolic regulation.

## 4. Mass Spectrometry Methods

One of the major challenges in workflows related to cell-type-specific proteomics is loss of protein during sample handling, which occurs at various steps between isolation of the single or multiple cell and peptide injection onto the mass spectrometer. Furthermore, enzymatic cleavage is necessary to generate peptides for bottom-up proteomics, but this can result in partial or incomplete digestion depending on the amino acid composition of the protein. Peptides generated from poor cleavage are often too large for ionization and detection via LC-MS/MS, ultimately resulting in loss of information for these specific regions of the protein. Instrument issues also include sensitivity and accuracy as well as chromatographic and spectral reproducibility between sample runs.

**Figure 2:**
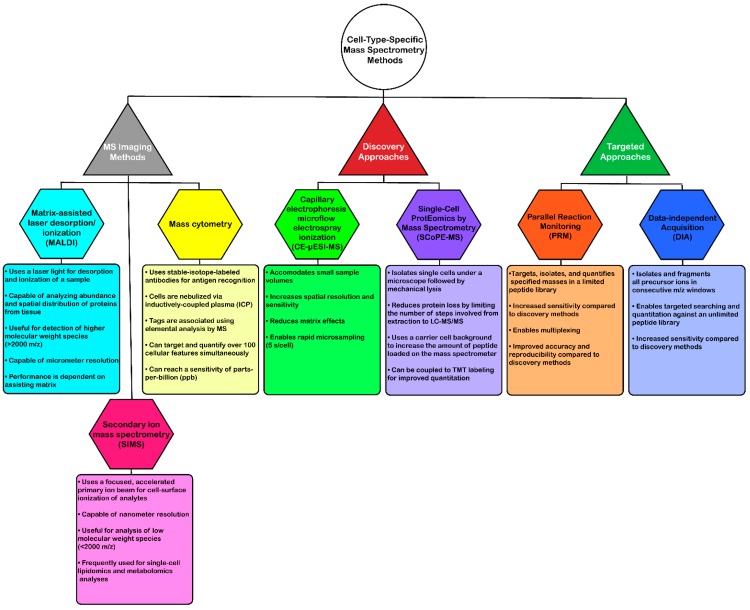
Overview of common mass spectrometry-based methods that are currently used for cell-type-specific analyses. Tree includes method type (triangles), name (hexagon) and a list of features associated with each method (rectangle).

Efforts to overcome some of these issues have utilized alternative workflows in an attempt to obtain cell-type level proteome or metabolome analysis (Figure 2). One such method termed mass spectrometry imaging (MSI) can analyze tissue sections with high spatial resolution to determine relative abundances and distribution of proteins [105,106,107,108,109,110,111]. Of the MS ionization sources available, matrix-assisted laser desorption/ionization (MALDI) and secondary ion mass spectrometry (SIMS) microprobes are most commonly used for imaging mass spectrometry due to their softer, non-destructive qualities that enable ionization of intact biomolecules at micro- and nanometer resolutions, respectively [105,112,113]. MALDI uses a laser light for desorption and ionization of the sample, and SIMS uses a more focused, accelerated primary ion beam to ionize analytes from the surface of cells. Furthermore, MALDI is particularly useful for detecting higher molecular weight species (2–70 kDa), while SIMS offers detection of molecules below 1 kDa or 2000 *m/z* [112,114,115,116].

These methods have been used for a range of experimental cell-type-specific applications [106,107,117,118,119,120,121]. For instance, MALDI-MSI was performed in mouse pituitary gland samples at a spatial resolution of 5 µm to identify ten neuropeptides at up to 2500 *m/z* [117]. An earlier study identified proteins in over 82 mass ranges in different mouse brain regions as well as 150 proteins in human glioblastoma tissue using MALDI-MSI [107]. One of the most recent MALDI-MSI applications demonstrated proteomic profiling of over 1000 rat dorsal root ganglia cells, which were classified into three separate groups on a peptide and lipid data basis [118]. SIMS has also been used for identification of single-cell metabolites, however, the majority of these studies focus on lipidomic analyses [120,121]. One study also used both SIMS and MALDI-MSI approaches to investigate the biomolecular and spatial composition of rat spinal cord tissue [116].

Mass cytometry is another type of MSI method that uses inductively coupled plasma (ICP) as an ionization source. This method is viewed as a targeted approach to MSI and uses metal-conjugated antibodies to enable antigen localization within the tissue or cell of interest, ultimately improving the limits of detection for target proteins. This multiplexing method enables quantitation of 100 target features, simultaneously without spectral overlap [122,123,124]. Bandura et al. [122] developed a 20-antigen targeted mass cytometry expression assay using lanthanide-tagged antibodies. This assay was then used to label cell lines from human leukemia patients (monoblastic M5 AML and monocytic M5 AML) and model cell lines (KG1a and Ramos) and subsequently map the isotope tag intensity profiles for an average of 15,000–20,000 cells [122]. A later report used bone marrow aspirates from a total of 46 leukemia and healthy patients to quantify 20 target biomarkers via mass cytometry [125]. Recently, tissue preparation techniques were compared for mass cytometry analysis of single-cell suspensions of human glioma, melanoma, and tonsil tissues [124]. A variation on this method was later developed, termed multiplexed ion beam imaging (MIBI), which images metal isotope-labeled antibodies using SIMS [123]. This method is also capable of imaging up to 100 features simultaneously at a parts-per-billion (ppb) sensitivity and is compatible with fixed tissue. Angelo et al. [123] used MIBI to quantify 10 biomarker targets in breast cancer biopsy tissue, which performed at the same level or better than other quantitative clinical immunohistochemistry (IHC) methods.

While there are clear advantages associated with MSI methods for single-cell proteomic and metabolic analyses, including sensitivity and multiplexing capabilities, there are still several drawbacks to these methods. As previously mentioned, MALDI-MSI is limited to higher molecular weight species (>2 kDa), while SIMS is limited to low molecular weight species (<2 kDa). Furthermore, MALDI is only capable of micrometer resolution and performance is dependent on the assisting matrix [105,112,113,126]. Mass cytometry is limited by the number of available metal-isotope-labeled antibodies and the specificity of the antibodies to the target antigen(s). Despite the possible disadvantages, advances in these mass spectrometry techniques have enormous potential to significantly improve the quality of data obtained from cell-type-specific proteomic analyses.

## 5. Future Perspectives

Cell-type-specific proteomics has undoubtedly made considerable progress in recent years, particularly in the field of neuroscience. Not only have cell isolation methods improved, but the instrumentation used for proteomic analysis has significantly advanced regarding sensitivity and reproducibility. Based on many of the neural cell-type-specific datasets available, however, the average number of proteins identified continues to fall far below the acceptable threshold of previous neural proteomics reports (Table A1) [90,91]. As discussed, there are several possible reasons for the discrepancy in protein identifications found in brain tissue versus single-cell datasets. One is the lack of organism- and tissue-specific standardization to determine the threshold of cellular material necessary for adequate proteomic analysis. As displayed in Table A1, the number of proteins identified in each of the listed techniques varies drastically between studies. Moreover, many of the results listed are lacking experimental information that is necessary for reproduction. For instance, several reports provide the number of cells and/or tissue quantity isolated but do not include the amount of protein extracted from this material or injected onto the mass spectrometer. This calls attention to the benefit of better standardization methods for cell-type-specific proteomics, in order to improve overall reproducibility and quality of datasets. Furthermore, method development for cell-type-specific proteomics in neuroscience needs to continue with increased focus placed on factors such as improving the efficiencies of cell isolation methods and reducing protein loss during sample preparation.

Recent efforts have also been made to improve these issues in the context of FACS for proteomic analysis. For instance, Zhu et al. [25] identified an average of 670 protein groups from single HeLa cells after integrating FACS and a novel method called nanoPOTS (nano-droplet processing in one-pot for trace samples). After cells are sorted via FACS, the nanoPOTS method relies on robotic liquid handling to perform sample processing in nanoliter volumes to help minimize sample loss. In this study, FACS was noted to have several advantages in a single-cell proteomic workflow such as precise cell counting and enabling removal of unwanted background contamination through cell dilution in PBS [25].

In addition to FACS-based approaches, development of mass spectrometry-based methods that combine different analytical features have made considerable progress in the advancement of single-cell proteomics. Capillary electrophoresis (CE) is one feature that has been recently coupled to mass spectrometry methods for single-cell analysis [127,128,129,130,131,132,133,134,135,136]. Benefits of using CE for single-cell analyses include small sample volume accommodation, increased spatial resolution and sensitivity, and reduced matrix effects [131,137,138,139]. One group recently coupled CE to microflow electrospray ionization mass spectrometry (CE-µESI-MS) to identify metabolites in different cell types of South African clawed frog (*Xenopus laevis*) embryos in three consecutive studies [129,130,131]. In the first of these studies, CE-µESI-MS was used to compare metabolites in three different *Xenopus* blastomere cell types dissected from the dorsal-ventral and animal-vegetal regions of the 16-cell embryo [130]. In total, 40 metabolites were significantly altered among the three cell types, indicating both specificity and metabolic interconnection. A year later, this group used a similar method to identify 55 unique small molecules in left and right D1 cells isolated from 8-cell *Xenopus* embryos [131]. After multivariate and statistical analyses, an equal number of five metabolites were found to be significantly enriched in the left and right D1 cells. Recently, this group was able to use CE-µESI-MS for direct analysis of live *Xenopus* embryo cells [129]. In this study, approximately 230 different molecular features were identified during mass spectrometry analysis of dorsal and ventral 8–32-cell-embryos. Not only has this group identified metabolites using CE-µESI-MS, but they have also performed proteomic analyses. In one report, they identified a total of 438 proteins from 16 ng of protein digest from a single blastomere of a *Xenopus* 16-cell embryo [132]. In the same year, they also reported identification of a total of 1709 protein groups from 20 ng of *Xenopus* protein digest from three cell types of the 16-cell embryo [133]. In addition to electrophoresis, capillaries have recently been used for microsampling of biomolecules from single neurons [140]. This study integrated this technique with downstream ESI-IMS-MS, which had only previously been performed in human carcinoma cells [141] and *Arabidopsis thaliana* epidermal cells [142]. Another study developed a neuron-in-capillary method to culture and isolate single *Aplysia californica* bag cell neurons prior to LC-MS/MS analysis [143].

Recently, a mass spectrometry-based approach called Single Cell ProtEomics by Mass Spectrometry (SCoPE-MS) was developed to address two of the major challenges facing cell-type-specific proteomic analysis: minimizing protein loss that can occur from protein extraction to mass spectrometry analysis and improving quantitation of low-abundant peptides identified from single cells [144]. To achieve these goals, live single mouse embryonic stem cells were isolated under a microscope prior to mechanical lysis and protein extraction. Next, single-cell protein was added to that of carrier cells to further reduce sample loss and increase the amount of protein injected on the mass spectrometer. To improve quantitation, tryptic peptides were then subjected to TMT labeling prior to LC-MS/MS, which resulted in quantitation of over 1000 proteins.

Despite the many advantages discovery mass spectrometry has to offer, more quantitative MS approaches have become increasingly popular in recent years. Targeted methods such as parallel reaction monitoring (PRM) and data-independent acquisition (DIA) have emerged in recent years in efforts to improve sensitive, accurate, and reproducible peptide quantitation. Though PRM is limited by the number of peptides that can be quantified in a given assay, it enables multiplexing, which can result in quantitation of multiple peptides in a single run for a more high-throughput analysis. Recently, Wan et al. [145] used PRM to quantify phosphorylation of PINK1 substrates in human and mouse cortical neurons. Data-independent acquisition (DIA) is not as sensitive as PRM, however, it has a much greater assay capacity. For instance, DIA analysis of fractionated mouse hippocampal neurons resulted in identification of 4558 proteins among all fractions [146]. A similar method to DIA was recently reported termed “BoxCar” which enabled identification of more than 10,000 proteins from mouse brain tissue [147]. Finally, label-based quantitation is another method that is becoming increasingly popular for neuroproteomic analyses. Recently, 11,840 protein groups were identified across two brain regions of control, AD, PD, and AD/PD human patients using TMT 10-plex labeling [148]. While these and other results mentioned above using LCM together with fixed tissue or MALDI-MSI are encouraging, there is a need for systematic and comprehensive cell-type-specific LC-MS-MS analyses in human tissue.

Targeted mass spectrometry is also useful for quantitation of protein isoforms, which can have cell-type- and tissue-specific expression profiles. Since the majority of isoform sequences are highly conserved, they can only be distinguished by isoform-specific peptides, which are often lower in abundance than peptides within the conserved regions. If these specific peptides are not detected via discovery LC-MS/MS, the isoforms cannot be distinguished and are consequently grouped by the mass spectrometry search software. This ultimately results in loss of isoform-specific expression profiles. Using a more sensitive targeted approach drastically improves the probability that isoform-specific peptides will be detected and quantifiable. Depending on the protein sequence, however, it may not be possible to identify specific peptides for all isoforms using the targeted mass spectrometry approach. One of the remaining ways to elucidate isoform-specific expression patterns is through mRNA sequencing. mRNA is alternatively spliced prior to protein translation and is therefore a blueprint for the protein sequence. By integrating the mRNA and protein datasets, a more complete picture of the proteome can be generated. Tools to achieve this type of data integration have already been developed, and continue to improve, which could prove useful for future cell-type-specific analyses [149,150].

In summary, there is an overwhelming demand for comprehensive and consistent cell-type-specific data in neuroscience, and novel techniques have been evolving rapidly in attempts to fill this gap. This review has outlined methods and technical challenges present in this area of research as well as potential improvements for these analyses. Collectively, these methods are making substantial progress to increase the sensitivity, reproducibility and depth of proteome coverage necessary for future cell-type-specific studies.

## Appendix A

**Table A1:** List of cell-type-specific methods for isolation, enrichment, and detection of proteins. The advantages and disadvantages of each technique are listed in Columns 1–3. References (Ref.) which have demonstrated the corresponding technique for a cell-type-specific application are listed in Column 4. Columns 5–8 contain the cell source (5), isolated cell/tissue quantity (6), protein quantity used for MS analysis (7), and number of proteins identified in the MS analysis (8) for each of the listed references. N/A indicates that information was not provided in the reference text.

Technique	Advantages	Disadvantages	Ref.	Cell Source	# Cells/Tissue Quantity Isolated	Protein Quantity for MS Analysis	# Proteins Identified from MS Analysis
Fluorescence-activated cell sorting (FACS)	Can purify functionally homogenous cell populations Offers precise cell counting Enables removal of background contamination	Cellular integrity can be compromised Low cell/protein yield if fluorescent expression/signal is low	[151]	Human neuronal nuclei	1 g starting tissue, >5 × 10^6^ nuclei	25 µg	1755
			[52]	Mouse inner ear hair cells	199,894 cells	3 µg	6333
			[51]	Mouse glutamatergic synapses	485 synapses	8 µg	2044 total, 163 enriched
			[25]	HeLa cells	1 cell	N/A	670
Laser-capture microdissection (LCM)	High-precision laser enables isolation of neurons (<100 µm^2^) Imaging and dissection can be performed in fluorescence or bright-field modes Compatible with fixed tissue	Tissue cannot be kept cold and may endure heat damage from the laser, potentially causing changes in protein expression or post-translational modifications Limited by the number of cells that can be analyzed per tissue slice Endogenous expression of fluorescent marker may not be adequate to visualize and dissect	[53]	Human cortical neurons from AD patients	4000–80,000 neurons	N/A	202 (4k neurons), 1773 (80k neurons)
			[54]	Human *substantia nigra*	550,000 µm^2^ neuromelanin (NM) granules	200 ng	1000
			[57]	Human neurons and blood brain barrier (BBB) structures	2500 neurons and 4000 BBB units	N/A	365 (Neurons), 539 (BBB)
			[26]	Rat cortical cells	2–6, 10–18, and 30–50 cells	N/A	180 (2–6 cells), 695 (10–18 cells), 1827 (30–50 cells)
			[152]	Human pancreatic islets	18 islets	N/A	3219
			[55]	FFPE fetal human brain tissue	36 samples (4 compartments, 8–15 mm^2^/compartment)	10 µg	3041
Induced pluripotent stem cells (iPSCs)	Resembles the human model more than commonly-used rodent models Can be differentiated into any cell type Less ethical challenges than embryonic cells	All analyses are in vitro Neural connectivity is lost	[70]	iPSCs	10^8^ cells	N/A	7952
			[69]		N/A	4 µg	9510
			[72]		N/A	40 µg	673
			[73]		6 × 10^4^ cells	240 µg	2217
			[74]		N/A	100 µg	1855
			[55]		2 × 10^7^ cells	10 µg	2875
BioOrthogonal Non-Canonical Amino Acid Tagging (BONCAT)	Enables in situ proteome labeling Enables time-dependent profiling of protein synthesis Non-canonical amino acid administration through drinking water or via injection Can be performed in fixed tissue	Metabolic incorporation needs to be performed in Met-free media or animals on a low Met diet Temporal resolution is limited by conversion of non-canonical amino acid into aminoacyl-tRNA prior to protein synthesis Labeled peptides are poorly detected with mass spectrometry Requires optimization of labeling efficiency	[84]	HEK293T cells	N/A	1.95–2.1 mg input	195
			[153]	HEK293T cells	N/A	N/A	138
			[85]	Excitatory hippocampal neurons, cerebellar Purkinje cells	130–200 k neurons (Purkinje)	N/A	2384 (hippocampal), 1687 (Purkinje)
				
Stochastic Orthogonal Recoding of Translation (SORT)	Viral-mediated expression of a modified tRNA (Does not require generation of a transgenic mouse) Can be performed in fixed tissue	Viral expression could be variable depending on the promoter used Optimization is required to determine time-dependent expression levels of tRNA synthase and labeling efficiency	[87]	Fly germ cells	500 ovaries	7 mg	299
			[89]	Mouse striatal medium spiny neurons (MSNs)	N/A	N/A	1780
Antibody-assisted cell-type-specific puromycylation	Does not require use of transgenic animal Displays high temporal resolution Functions at lower concentrations than noncanonical amino acids	Relies on antibody specificity	[94]	A431 cells	N/A	N/A	>1200
			[95]	HEK293T cells	2 × 10^7^ cells	N/A	1165 enriched
					
BioID	Enables screening of proximal protein interactors in situ	Time-consuming (Need to generate and characterize transgenic mice) Extensive assay optimization required Labeling times are slow (~24 h)	[97]	HEK293T cells	4 × 10^7^ cells	N/A	122
			[154]	*Toxoplasma gondii* parasite	N/A	N/A	19
			[98]	Mouse cortical and hippocampal neurons	N/A	N/A	121 (ePSD), 181 (iPSD)
BioID2	Uses a smaller biotin ligase than BioID Enables more selective targeting of fusion proteins than BioID Requires less biotin supplementation than BioID Displays enhanced labeling of proximal interacting proteins than BioID	Time-consuming (Need to generate and characterize transgenic mice) Extensive assay optimization required Labeling times are moderately slow (~16 h)	[99]	HEK293T cells	4 × 10^7^ cells	100 µg	260
				
				
				
TurboID	Efficient labeling time (~10 min) Compatible with TMT labeling Enables labeling of organelle-specific proteomes	Can sequester endogenous biotin and cause toxicity	[100]	HEK293T cells	N/A	3 mg input	314 (mito), 186 (ER), 1455 (nuclear)
		Long labeling times can cause toxicity					
					
					
Engineered ascorbate peroxidase (APEX)	Enables screening of proximal protein interactors in situ Labeling is very rapid (~1 min) Applicable for labeling of subcellular compartments	Limited stability in heated or reducing environments Generating a transgenic organism is necessary H2O2 can cause cellular toxicity	[101]	HEK293T cells	7–8 million cells	4 mg input	495
			[155]	*Drosophila melanogaster*	N/A	N/A	389
			[102]	*C. elegans* L4 larvae	30,000 larval cells	450–500 µg input	3180
Matrix-assisted laser desorption/ionization MS imaging (MALDI-MSI)	Enables spatial quantitation of proteins in tissue sections	Low spatial resolution (µm) Broad mass range (~500–100 kDa)	[107]	APP23 transgenic mouse tissue	50 µm resolution	N/A	5 Aβ peptides
	Non-destructive method		[116]	Rat spinal cord	20 µm tissue sections	N/A	27 peptides
			[117]	Mouse pituitary gland	1.5 mm × 2.5 mm tissue sections	N/A	10 neuropeptides
			[118]	Rat dorsal root ganglia	>1000 cells	N/A	26 peptides
Secondary ion mass spectrometry (SIMS)	Enables spatial quantitation of proteins in tissue sections Non-destructive method	High spatial resolution (nm) Low mass range (<1000 Da)	[121]	Benign prostatic hyperplasia (BPH), HeLa, and human cheek cells	25–30 µm diameter tissue (BPH: 180 × 180 µm^2^, HeLa: 88 × 108 µm^2^, cheek cells: 150 × 175 µm^2^)	N/A	<10 biomolecule ions
			[116]	Rat spinal cord	2.3 µm spatial resolution	N/A	18 biomolecule ions
			[120]	*Aplysia californica* neurons	0.39–2.3 µm resolution	N/A	3 biomolecule ions
Mass cytometry	Enables multiplexed targeting of 100 target features without spectral overlap	Limited by the number and specificity of available metal-isotope-labeled antibodies	[122]	Human leukemia cells (monoblastic M5 AML, monocytic M5 AML) and model cell lines (KG1a, Ramos)	15,000–20,000 cells	N/A	20 target antigens
			[123]	Human breast tumor cells	N/A	N/A	10 target antigens
			[125]	Human bone marrow aspirates	480,000 cells	N/A	28 target antigens
			[124]	Human glioma, melanoma, and tonsil tissue cells	N/A	N/A	8 target antigens
Capillary electrophoresis microflow electrospray ionization mass spectrometry (CE-µESI-MS)	Accommodates small sample volumes High spatial resolution and sensitivity Low matrix effects Can be temperature controlled to avoid sample heating	Extensive optimization required	[143]	*Aplysia californica* neurons	1 neuron	N/A	>300 metabolites
			[127]	*Aplysia californica* neurons	25 B1 and B2 buccal neurons	N/A	>300 metabolites
			[130]	*Xenopus laevis* 16-cell embryo	15 blastomeres	N/A	40 metabolites
			[131]	*Xenopus laevis* 8-cell embryo	D1 blastomere	N/A	55 small molecules
			[132]	*Xenopus laevis* 16-cell embryo	1 blastomere	16 ng	438
			[133]	*Xenopus laevis* 16-cell embryo	1 blastomere	20 ng	500–800
			[129]	*Xenopus laevis* 8–32-cell embryo	1 blastomere	N/A	230 molecular features
Single Cell ProtEomics by Mass Spectrometry (SCoPE-MS)	Minimizes protein loss from protein extraction to LC-MS/MS Quantitative MS approach (TMT labeling)	Has been demonstrated in few organisms	[144]	Mouse embryonic stem cells	1 cell		>1000 proteins
				

## References

[1] Kitchen R.R., Rozowsky J.S., Gerstein M.B., Nairn A.C. (2014). Decoding neuroproteomics: Integrating the genome, translatome and functional anatomy. Nat. Neurosci..

[2] Herculano-Houzel S. (2014). The glia/neuron ratio: How it varies uniformly across brain structures and species and what that means for brain physiology and evolution. Glia.

[3] Herculano-Houzel S., Lent R. (2005). Isotropic Fractionator: A Simple, Rapid Method for the Quantification of Total Cell and Neuron Numbers in the Brain. J. Neurosci..

[4] Castelo-Branco G., Sousa K.M., Bryja V., Pinto L., Wagner J., Arenas E. (2005). Ventral midbrain glia express region-specific transcription factors and regulate dopaminergic neurogenesis through Wnt-5a secretion. Mol. Cell. Neurosci..

[5] Crompton L.A., Cordero-Llana O., Caldwell M.A. (2017). Astrocytes in a dish: Using pluripotent stem cells to model neurodegenerative and neurodevelopmental disorders. Brain Pathol..

[6] Angerer P., Simon L., Tritschler S., Wolf F.A., Fischer D., Theis F.J. (2017). Single cells make big data: New challenges and opportunities in transcriptomics. Curr. Opin. Syst. Biol..

[7] Smith R.D., Malley J.D., Schechter A.N. (2000). Quantitative analysis of globin gene induction in single human erythroleukemic cells. Nucleic Acids Res..

[8] Bengtsson M., Ståhlberg A., Rorsman P., Kubista M. (2005). Gene expression profiling in single cells from the pancreatic islets of Langerhans reveals lognormal distribution of mRNA levels. Genome Res..

[9] Levsky J.M., Shenoy S.M., Pezo R.C., Singer R.H. (2002). Single-Cell Gene Expression Profiling. Science.

[10] Tirosh I., Venteicher A.S., Hebert C., Escalante L.E., Patel A.P., Yizhak K., Fisher J.M., Rodman C., Mount C., Filbin M.G. (2016). Single-cell RNA-seq supports a developmental hierarchy in human oligodendroglioma. Nature.

[11] Hashimshony T., Wagner F., Sher N., Yanai I. (2012). CEL-Seq: Single-Cell RNA-Seq by Multiplexed Linear Amplification. Cell Rep..

[12] Ziegenhain C., Vieth B., Parekh S., Reinius B., Guillaumet-Adkins A., Smets M., Leonhardt H., Heyn H., Hellmann I., Enard W. (2017). Comparative Analysis of Single-Cell RNA Sequencing Methods. Mol. Cell.

[13] Zeisel A., Muñoz-Manchado A.B., Codeluppi S., Lönnerberg P., La Manno G., Juréus A., Marques S., Munguba H., He L., Betsholtz C. (2015). Cell types in the mouse cortex and hippocampus revealed by single-cell RNA-seq. Science.

[14] Lake B., Shen R., Ronaghi M., Fan J., Wang W., Zhang K. (2016). Neuronal subtypes and diverstiy revealed by single-nucleus RNA sequencing of human brain. Science.

[15] Han X., Wang R., Zhou Y., Fei L., Sun H., Lai S., Saadatpour A., Zhou Z., Chen H., Ye F. (2018). Mapping the Mouse Cell Atlas by Microwell-Seq. Cell.

[16] Macosko E.Z., Basu A., Satija R., Nemesh J., Shekhar K., Goldman M., Tirosh I., Bialas A.R., Kamitaki N., Martersteck E.M. (2015). Highly Parallel Genome-wide Expression Profiling of Individual Cells Using Nanoliter Droplets. Cell.

[17] Klein A.M., Mazutis L., Akartuna I., Tallapragada N., Veres A., Li V., Peshkin L., Weitz D.A., Kirschner M.W. (2015). Droplet barcoding for single-cell transcriptomics applied to embryonic stem cells. Cell.

[18] Saunders A., Macosko E.Z., Wysoker A., Goldman M., Krienen F.M., de Rivera H., Bien E., Baum M., Bortolin L., Wang S. (2018). Molecular Diversity and Specializations among the Cells of the Adult Mouse Brain. Cell.

[19] Zeisel A., Hochgerner H., Lönnerberg P., Johnsson A., Memic F., van der Zwan J., Häring M., Braun E., Borm L.E., La Manno G. (2018). Molecular Architecture of the Mouse Nervous System. Cell.

[20] Liu Y., Beyer A., Aebersold R. (2016). On the Dependency of Cellular Protein Levels on mRNA Abundance. Cell.

[21] Vidova V., Spacil Z. (2017). A review on mass spectrometry-based quantitative proteomics: Targeted and data independent acquisition. Anal. Chim. Acta.

[22] Eliuk S., Makarov A. (2015). Evolution of Orbitrap Mass Spectrometry Instrumentation. Annu. Rev. Anal. Chem..

[23] Sinitcyn P., Daniel Rudolph J., Cox J. (2018). Computational Methods for Understanding Mass Spectrometry-Based Shotgun Proteomics Data. Annu. Rev. Biomed. Data Sci..

[24] O’Connell J.D., Paulo J.A., O’Brien J.J., Gygi S.P. (2018). Proteome-Wide Evaluation of Two Common Protein Quantification Methods. J. Proteome Res..

[25] Zhu Y., Clair G., Chrisler W.B., Shen Y., Zhao R., Shukla A.K., Moore R.J., Misra R.S., Pryhuber G.S., Smith R.D. (2018). Proteomic analysis of single mammalian cells enabled by microfluidic nanodroplet sample preparation and ultrasensitive nanoLC-MS. Angew. Chem. Int. Ed..

[26] Zhu Y., Dou M., Piehowski P.D., Liang Y., Wang F., Chu R.K., Chrisler W.B., Smith J.N., Schwarz K.C., Shen Y. (2018). Spatially resolved proteome mapping of laser capture microdissected tissue with automated sample transfer to nanodroplets Running Title: Spatially-resolved proteomics using nanoPOTS platform. Mol. Cell. Proteom..

[27] Bertran-Gonzalez J., Bosch C., Maroteaux M., Matamales M., Hervé D., Valjent E., Girault J.-A. (2008). Opposing Patterns of Signaling Activation in Dopamine D1 and D2 Receptor-Expressing Striatal Neurons in Response to Cocaine and Haloperidol. J. Neurosci..

[28] Clark D., White F.J. (1997). D1 dopamine receptor - the search for a function: A critical evaluation of the D1/D2 dopamine classification and its functional implications. Synapse.

[29] Bateup H.S., Svenningsson P., Kuroiwa M., Gong S., Nishi A., Heintz N., Greengard P. (2008). Cell type-specific regulation of DARPP-32 phosphorylation by psychostimulant and antipsychotic drugs. Nat. Neurosci..

[30] Braak H., Braak E. (1995). Staging of alzheimer’s disease-related neurofibrillary changes. Neurobiol. Aging.

[31] Braak H., Braak E. (1991). Neuropathological stageing of Alzheimer-related changes. Acta Neuropathol..

[32] Wyss-Coray T., Loike J.D., Brionne T.C., Lu E., Anankov R., Yan F., Silverstein S.C., Husemann J. (2003). Adult mouse astrocytes degrade amyloid-β in vitro and in situ. Nat. Med..

[33] Chun H., Lee C.J. (2018). Reactive astrocytes in Alzheimer’s disease: A double-edged sword. Neurosci. Res..

[34] Jo S., Yarishkin O., Hwang Y.J., Chun Y.E., Park M., Woo D.H., Bae J.Y., Kim T., Lee J., Chun H. (2014). GABA from reactive astrocytes impairs memory in mouse models of Alzheimer’s disease. Nat. Med..

[35] Serrano-Pozo A., Muzikansky A., Gómez-Isla T., Growdon J.H., Betensky R.A., Frosch M.P., Hyman B.T. (2013). Differential Relationships of Reactive Astrocytes and Microglia to Fibrillar Amyloid Deposits in Alzheimer Disease. J. Neuropathol. Exp. Neurol..

[36] Itagaki S., Mcgeer P.L., Akiyama H., Zhu S., Selkoe D. (1989). Relationship of microglia and astrocytes to amyloid deposits of Alzheimer disease. J. Neuroimmunol..

[37] Spillantini M.G., Schmidt M.L., Lee V.M.-Y., Trojanowski J.Q., Jakes R., Goedert M. (1997). alpha-Synuclein in Lewy bodies. Nature.

[38] Brichta L., Shin W., Jackson-Lewis V., Blesa J., Yap E.L., Walker Z., Zhang J., Roussarie J.P., Alvarez M.J., Califano A. (2015). Identification of neurodegenerative factors using translatome-regulatory network analysis. Nat. Neurosci..

[39] Zhai S., Tanimura A., Graves S.M., Shen W., Surmeier D.J. (2018). Striatal synapses, circuits, and Parkinson’s disease. Curr. Opin. Neurobiol..

[40] Mallet N., Ballion B., Le Moine C., Gonon F. (2006). Cortical Inputs and GABA Interneurons Imbalance Projection Neurons in the Striatum of Parkinsonian Rats. J. Neurosci..

[41] Kravitz A.V., Freeze B.S., Parker P.R.L., Kay K., Thwin M.T., Deisseroth K., Kreitzer A.C. (2010). Regulation of parkinsonian motor behaviours by optogenetic control of basal ganglia circuitry. Nature.

[42] Kiernan M.C., Vucic S., Cheah B.C., Turner M.R., Eisen A., Hardiman O., Burrell J.R., Zoing M.C. (2011). Amyotrophic lateral sclerosis. Lancet.

[43] Rowland L.P., Shneider N.A. (2001). Amyotrophic Lateral Sclerosis. N. Engl. J. Med..

[44] Faideau M., Kim J., Cormier K., Gilmore R., Welch M., Auregan G., Dufour N., Guillermier M., Brouillet E., Hantraye P. (2010). In vivo expression of polyglutamine-expanded huntingtin by mouse striatal astrocytes impairs glutamate transport: A correlation with Huntington’s disease subjects. Hum. Mol. Genet..

[45] Santhakumar V., Jones R.T., Mody I. (2010). Developmental regulation and neuroprotective effects of striatal tonic GABAA currents. Neuroscience.

[46] Gong S., Zheng C., Doughty M.L., Losos K., Didkovsky N., Schambra U.B., Nowak N.J., Joyner A., Leblanc G., Hatten M.E. (2003). A gene expression atlas of the central nervous system based on artificial chromosomes. Nature.

[47] Heiman M., Schaefer A., Gong S., Peterson J.D., Day M., Ramsey K.E., Suá Rez-Fariñ M., Schwarz C., Stephan D.A., Surmeier D.J. (2008). A Translational Profiling Approach for the Molecular Characterization of CNS Cell Types. Cell.

[48] Lobo M.K., Karsten S.L., Gray M., Geschwind D.H., Yang X.W. (2006). FACS-array profiling of striatal projection neuron subtypes in juvenile and adult mouse brains. Nat. Neurosci..

[49] Marion-Poll L., Montalban E., Munier A., Hervé D., Girault J.A. (2014). Fluorescence-activated sorting of fixed nuclei: A general method for studying nuclei from specific cell populations that preserves post-translational modifications. Eur. J. Neurosci..

[50] Jordi E., Heiman M., Marion-Poll L., Guermonprez P., Cheng S.K., Nairn A.C., Greengard P., Girault J.-A. (2013). Differential effects of cocaine on histone posttranslational modifications in identified populations of striatal neurons. Proc. Natl. Acad. Sci. USA.

[51] Biesemann C., Grønborg M., Luquet E., Wichert S.P., Eronique Bernard V., Bungers S.R., Cooper B., Ed Erique Varoqueaux F., Li L., Byrne J.A. (2014). Proteomic screening of glutamatergic mouse brain synaptosomes isolated by fluorescence activated sorting. EMBO J..

[52] Hickox A.E., Wong A.C.Y., Pak K., Strojny C., Ramirez M., Yates J.R., Ryan A.F., Savas J.N. (2016). Global Analysis of Protein Expression of Inner Ear Hair Cells. J. Neurosci..

[53] Drummond E.S., Nayak S., Ueberheide B., Wisniewski T., Huang T.T. (2015). Proteomic analysis of neurons microdissected from formalin-fixed, paraffin-embedded Alzheimer’s disease brain tissue. Sci. Rep..

[54] Plum S., Steinbach S., Attems J., Keers S., Riederer P., Gerlach M., May C., Marcus K. (2016). Proteomic characterization of neuromelanin granules isolated from human substantia nigra by laser-microdissection. Sci. Rep..

[55] Djuric U., Rodrigues D.C., Batruch I., Ellis J., Shannon P., Diamandis P. (2017). Spatiotemporal proteomic profiling of human cerebral development. Mol. Cell. Proteom..

[56] Hondius D.C., Eigenhuis K.N., Morrema T.H.J., Van Der Schors R.C., Van Nierop P., Bugiani M., Li K.W., Hoozemans J.J.M., Smit A.B., Rozemuller A.J.M. (2018). Proteomics analysis identifies new markers associated with capillary cerebral amyloid angiopathy in Alzheimer’s disease. Acta Neuropathol. Commun..

[57] García-Berrocoso T., Llombart V., Colàs-Campàs L., Hainard A., Licker V., Penalba A., Ramiro L., Simats A., Bustamante A., Martínez-Saez E. (2018). Single Cell Immuno-Laser Microdissection Coupled to Label-Free Proteomics to Reveal the Proteotypes of Human Brain Cells After Ischemia. Mol. Cell. Proteom..

[58] Tagawa K., Homma H., Saito A., Fujita K., Chen X., Imoto S., Oka T., Ito H., Motoki K., Yoshida C. (2015). Comprehensive phosphoproteome analysis unravels the core signaling network that initiates the earliest synapse pathology in preclinical Alzheimer’s disease brain. Hum. Mol. Genet..

[59] Oka T., Tagawa K., Ito H., Okazawa H. (2011). Dynamic changes of the phosphoproteome in postmortem mouse brains. PLoS ONE.

[60] Li J., Gould T.D., Yuan P., Manji H.K., Chen G. (2003). Post-mortem Interval Effects on the Phosphorylation of Signaling Proteins. Neuropsychopharmacology.

[61] O’Callaghan J.P., Sriram K. (2004). Focused microwave irradiation of the brain preserves in vivo protein phosphorylation: Comparison with other methods of sacrifice and analysis of multiple phosphoproteins. J. Neurosci. Methods.

[62] Takahashi K., Tanabe K., Ohnuki M., Narita M., Ichisaka T., Tomoda K., Yamanaka S. (2007). Induction of Pluripotent Stem Cells from Adult Human Fibroblasts by Defined Factors. Cell.

[63] Takahashi K., Yamanaka S. (2006). Induction of Pluripotent Stem Cells from Mouse Embryonic and Adult Fibroblast Cultures by Defined Factors. Cell.

[64] Paolo F., Giorgio D., Boulting G.L., Bobrowicz S., Eggan K.C. (2008). Cell Stem Cell Human Embryonic Stem Cell-Derived Motor Neurons Are Sensitive to the Toxic Effect of Glial Cells Carrying an ALS-Causing Mutation. Stem Cell.

[65] Krencik R., Weick J.P., Liu Y., Zhang Z.-J., Zhang S.-C. (2011). Specification of transplantable astroglial subtypes from human pluripotent stem cells. Nat. Biotechnol..

[66] Kriks S., Shim J.-W., Piao J., Ganat Y.M., Wakeman D.R., Xie Z., Carrillo-Reid L., Auyeung G., Antonacci C., Buch A. (2011). Dopamine neurons derived from human ES cells efficiently engraft in animal models of Parkinson’s disease. Nature.

[67] Liu H., Zhang S.-C. (2011). Specification of neuronal and glial subtypes from human pluripotent stem cells. Cell. Mol. Life Sci..

[68] Shi Y., Kirwan P., Smith J., Maclean G., Orkin S.H., Livesey F.J. (2012). A human stem cell model of early Alzheimer’s disease pathology in Down syndrome. Sci. Transl. Med..

[69] Yamana R., Iwasaki M., Wakabayashi M., Nakagawa M., Yamanaka S., Ishihama Y. (2013). Rapid and Deep Profiling of Human Induced Pluripotent Stem Cell Proteome by One-shot NanoLC−MS/MS Analysis with Meter-scale Monolithic Silica Columns. J. Proteome Res..

[70] Phanstiel D.H., Brumbaugh J., Wenger C.D., Tian S., Bolin J.M., Ruotti V., Stewart R., Thomson J.A., Coon J.J. (2012). Proteomic and phosphoproteomic comparison of human ES and iPS cells. Nat. Methods.

[71] Chae J.-I., Kim D.-W., Lee N., Jeon Y.-J., Jeon I., Kwon J., Kim J., Soh Y., Lee D.-S., Kang S. (2012). Quantitative proteomic analysis of induced pluripotent stem cells derived from a human Huntington’s disease patient. Biochem. J.

[72] Hao J., Li W., Dan J., Ye X., Wang F., Zeng X., Wang L., Wang H., Cheng Y., Liu L. (2013). Reprogramming- and pluripotency-associated membrane proteins in mouse stem cells revealed by label-free quantitative proteomics. J. Proteom..

[73] Fuller H.R., Mandefro B., Shirran S.L., Gross A.R., Kaus A.S., Botting C.H., Morris G.E., Sareen D. (2016). Spinal Muscular Atrophy Patient iPSC-Derived Motor Neurons Have Reduced Expression of Proteins Important in Neuronal Development. Front. Cell. Neurosci..

[74] Chen M., Lee H.-K., Moo L., Hanlon E., Stein T., Xia W. (2018). Common proteomic profiles of induced pluripotent stem cell-derived three-dimensional neurons and brain tissue from Alzheimer patients. J. Proteom..

[75] Thompson A., Schäfer J., Kuhn K., Kienle S., Schwarz J., Schmidt G., Neumann T., Hamon C. (2003). Tandem mass tags: A novel quantification strategy for comparative analysis of complex protein mixtures by MS/MS. Anal. Chem..

[76] Ong S.-E., Blagoev B., Kratchmarova I., Kristensen D.B., Steen H., Pandey A., Mann M. (2002). Stable Isotope Labeling by Amino Acids in Cell Culture, SILAC, as a Simple and Accurate Approach to Expression Proteomics. Mol. Cell. Proteom..

[77] Mann M. (2006). Functional and quantitative proteomics using SILAC. Nat. Rev. Mol. Cell Biol..

[78] Schwanhäusser B., Gossen M., Dittmar G., Selbach M. (2009). Global analysis of cellular protein translation by pulsed SILAC. Proteomics.

[79] de Godoy L.M.F., Olsen J.V., de Souza G.A., Li G., Mortensen P., Mann M. (2006). Status of complete proteome analysis by mass spectrometry: SILAC labeled yeast as a model system. Genome Biol..

[80] Zhang A., Uaesoontrachoon K., Shaughnessy C., Das J.R., Rayavarapu S., Brown K.J., Ray P.E., Nagaraju K., van den Anker J.N., Hoffman E.P. (2015). The use of urinary and kidney SILAM proteomics to monitor kidney response to high dose morpholino oligonucleotides in the mdx mouse. Toxicol. Rep..

[81] McClatchy D.B., Liao L., Park S.K., Xu T., Lu B., Yates J.R. (2011). Differential proteomic analysis of mammalian tissues using SILAM. PLoS ONE.

[82] Mcclatchy D.B., Liao L., Park S.K., Venable J.D., Yates J.R. (2007). Quantification of the synaptosomal proteome of the rat cerebellum during post-natal development. Genome Res..

[83] Rauniyar N., McClatchy D.B., Yates J.R. (2013). Stable isotope labeling of mammals (SILAM) for in vivo quantitative proteomic analysis. Methods.

[84] Dieterich D.C., Link A.J., Graumann J., Tirrell D.A., Schuman E.M., Sharpless K.B. (2006). Selective identification of newly synthesized proteins in mammalian cells using bioorthogonal noncanonical amino acid tagging (BONCAT). Proc. Natl. Acad. Sci. USA.

[85] Alvarez-Castelao B., Schanzenbächer C.T., Hanus C., Glock C., Tom Dieck S., Dörrbaum A.R., Bartnik I., Nassim-Assir B., Ciirdaeva E., Mueller A. (2017). Cell-type-specific metabolic labeling of nascent proteomes in vivo. Nat. Biotechnol..

[86] Dieterich D.C., Hodas J.J.L., Gouzer G., Shadrin I.Y., Ngo J.T., Triller A., Tirrell D.A., Schuman E.M. (2010). In situ visualization and dynamics of newly synthesized proteins in rat hippocampal neurons. Nat. Neurosci..

[87] Elliott T.S., Bianco A., Townsley F.M., Fried S.D., Chin J.W. (2016). Tagging and Enriching Proteins Enables Cell-Specific Proteomics. Cell Chem. Biol..

[88] Elliott T.S., Townsley F.M., Bianco A., Ernst R.J., Sachdeva A., Elsässer S.J., Davis L., Lang K., Pisa R., Greiss S. (2014). Proteome labeling and protein identification in specific tissues and at specific developmental stages in an animal. Nat. Biotechnol..

[89] Krogager T.P., Ernst R.J., Elliott T.S., Calo L., Beránek V., Ciabatti E., Spillantini M.G., Tripodi M., Hastings M.H., Chin J.W. (2018). Labeling and identifying cell-specific proteomes in the mouse brain. Nat. Biotechnol..

[90] Sharma K., Schmitt S., Bergner C.G., Tyanova S., Kannaiyan N., Manrique-Hoyos N., Kongi K., Cantuti L., Hanisch U.-K., Philips M.-A. (2015). Cell type-and brain region-resolved mouse brain proteome. Nat. Neurosci..

[91] Carlyle B.C., Kitchen R.R., Kanyo J.E., Voss E.Z., Pletikos M., Sousa A.M.M., Lam T.T., Gerstein M.B., Sestan N., Nairn A.C. (2017). A Multiregional Proteomic Survey of the Postnatal Human Brain. Nat. Neurosci..

[92] Liu J., Xu Y., Stoleru D., Salic A. (2012). Imaging protein synthesis in cells and tissues with an alkyne analog of puromycin. Proc. Natl. Acad. Sci. USA.

[93] Ge J., Zhang C.W., Ng X.W., Peng B., Pan S., Du S., Wang D., Li L., Lim K.L., Wohland T. (2016). Puromycin Analogues Capable of Multiplexed Imaging and Profiling of Protein Synthesis and Dynamics in Live Cells and Neurons. Angew. Chem. Int. Ed..

[94] Du S., Wang D., Lee J.-S., Peng B., Ge J., Yao S. (2017). Cell Type-Selective Imaging and Profiling of Newly Synthesized Proteomes by Using Puromycin Analogues. Chem. Commun..

[95] Barrett R.M., Liu H.W., Jin H., Goodman R.H., Cohen M.S. (2016). Cell-specific Profiling of Nascent Proteomes Using Orthogonal Enzyme-mediated Puromycin Incorporation. ACS Chem. Biol..

[96] Li Z., Zhu Y., Sun Y., Qin K., Liu W., Zhou W., Chen X. (2016). Nitrilase-Activatable Noncanonical Amino Acid Precursors for Cell-Selective Metabolic Labeling of Proteomes. ACS Chem. Biol..

[97] Roux K.J., Kim D.I., Raida M., Burke B. (2012). A promiscuous biotin ligase fusion protein identifies proximal and interacting proteins in mammalian cells. J. Cell Biol..

[98] Uezu A., Kanak D.J., Bradshaw T.W.A., Soderblom E.J., Catavero C.M., Burette A.C., Weinberg R.J., Soderling S.H. (2016). Identification of an elaborate complex mediating postsynaptic inhibition. Science.

[99] Kim D.I., Jensen S.C., Noble K.A., KC B., Roux K.H., Motamedchaboki K., Roux K.J. (2016). An improved smaller biotin ligase for BioID proximity labeling. Mol. Biol. Cell.

[100] Branon T.C., Bosch J.A., Sanchez A.D., Udeshi N.D., Svinkina T., Carr S.A., Feldman J.L., Perrimon N., Ting A.Y. (2018). Efficient proximity labeling in living cells and organisms with TurboID. Nat. Biotechnol..

[101] Rhee H.-W., Zou P., Udeshi N.D., Martell J.D., Mootha V.K., Carr S.A., Ting A.Y. (2013). Proteomic Mapping of Mitochondria in Living Cells via Spatially- Restricted Enzymatic Tagging. Science.

[102] Reinke A.W., Mak R., Troemel E.R., Bennett E.J. (2017). In vivo mapping of tissue-and subcellular-specific proteomes in Caenorhabditis elegans. Sci. Adv..

[103] Lobingier B.T., Hüttenhain R., Eichel K., Miller K.B., Ting A.Y., von Zastrow M., Krogan N.J. (2017). An Approach to Spatiotemporally Resolve Protein Interaction Networks in Living Cells. Cell.

[104] Loh K.H., Stawski P.S., Draycott A.S., Udeshi N.D., Lehrman E.K., Wilton D.K., Svinkina T., Deerinck T.J., Ellisman M.H., Stevens B. (2015). HHS Public Access. Cell.

[105] Comi T.J., Do T.D., Rubakhin S.S., Sweedler J.V. (2017). Categorizing Cells on the Basis of their Chemical Profiles: Progress in Single-Cell Mass Spectrometry. J. Am. Chem. Soc..

[106] Stoeckli M., Chaurand P., Hallahan D.E., Caprioli R.M. (2001). Imaging mass spectrometry: A new technology for the analysis of protein expression in mammalian tissues. Nat. Med..

[107] Stoeckli M., Staab D., Staufenbiel M., Wiederhold K.-H., Signor L. (2002). Molecular imaging of amyloid b peptides in mouse brain sections using mass spectrometry. Anal. Biochem..

[108] Schwamborn K., Caprioli R.M. (2010). Molecular imaging by mass spectrometry—Looking beyond classical histology. Nat. Rev. Cancer.

[109] Bozdon-Kulakowska A., Suder P. (2016). Imaging mass specrometry: Instrumentation, applications, and combination with other visualization techniques. Mass Spectrom. Rev..

[110] Rocha B., Ruiz-Romero C., Blanco F.J. (2016). Mass spectrometry imaging: A novel technology in rheumatology. Nat. Rev. Rheumatol..

[111] Spengler B. (2015). Mass Spectrometry Imaging of Biomolecular Information. Anal. Chem..

[112] Reyzer M.L., Caprioli R.M. (2005). MALDI Mass Spectrometry for Direct Tissue Analysis: A New Tool for Biomarker Discovery. J. Proteome Res..

[113] Giesen C., Wang H.A.O., Schapiro D., Zivanovic N., Jacobs A., Hattendorf B., Schüffler P.J., Grolimund D., Buhmann J.M., Brandt S. (2014). Highly multiplexed imaging of tumor tissues with subcellular resolution by mass cytometry. Nat. Methods.

[114] Karas M., Hillenkamp F. (1988). Laser desorption ionization of proteins with molecular masses exceeding 10,000 daltons. Anal. Chem..

[115] Zhang L., Vertes A. (2018). Single-Cell Mass Spectrometry Approaches to Explore Cellular Heterogeneity. Angew. Chem. Int. Ed..

[116] Monroe E.B., Annangudi S.P., Hatcher N.G., Gutstein H.B., Rubakhin S.S., Sweedler J.V. (2008). SIMS and MALDI MS imaging of the spinal cord. Proteomics.

[117] Guenther S., Römpp A., Kummer W., Spengler B. (2011). AP-MALDI imaging of neuropeptides in mouse pituitary gland with 5μm spatial resolution and high mass accuracy. Int. J. Mass Spectrom..

[118] Do T.D., Ellis J.F., Neumann E.K., Comi T.J., Tillmaand E.G., Lenhart A.E., Rubakhin S.S., Sweedler J.V. (2018). Optically Guided Single Cell Mass Spectrometry of Rat Dorsal Root Ganglia to Profile Lipids, Peptides and Proteins. ChemPhysChem.

[119] Do T.D., Comi T.J., Dunham S.J.B., Rubakhin S.S., Sweedler J.V. (2017). Single Cell Profiling Using Ionic Liquid Matrix-Enhanced Secondary Ion Mass Spectrometry for Neuronal Cell Type Differentiation. Anal. Chem..

[120] Tucker K.R., Li Z., Rubakhin S.S., Sweedler J.V. (2012). Secondary Ion Mass Spectrometry Imaging of Molecular Distributions in Cultured Neurons and Their Processes: Comparative Analysis of Sample Preparation. J. Am. Soc. Mass Spectrom..

[121] Fletcher J.S., Rabbani S., Henderson A., Blenkinsopp P., Thompson S.P., Lockyer N.P., Vickerman J.C. (2008). A New Dynamic in Mass Spectral Imaging of Single Biological Cells. Anal. Chem..

[122] Bandura D.R., Baranov V.I., Ornatsky O.I., Antonov A., Kinach R., Lou X., Pavlov S., Vorobiev S., Dick J.E., Tanner S.D. (2009). Mass Cytometry: Technique for Real Time Single Cell Multitarget Immunoassay Based on Inductively Coupled Plasma Time-of-Flight Mass Spectrometry. Anal. Chem..

[123] Angelo M., Bendall S.C., Finck R., Hale M.B., Hitzman C., Borowsky A.D., Levenson R.M., Lowe J.B., Liu S.D., Zhao S. (2014). Multiplexed ion beam imaging of human breast tumors. Nat. Med..

[124] Leelatian N., Doxie D.B., Greenplate A.R., Mobley B.C., Lehman J.M., Sinnaeve J., Kauffmann R.M., Werkhaven J.A., Mistry A.M., Weaver K.D. (2017). Single cell analysis of human tissues and solid tumors with mass cytometry. Cytom. Part B Clin. Cytom..

[125] Behbehani G.K., Samusik N., Bjornson Z.B., Fantl W.J., Medeiros B.C., Nolan G.P. (2015). Mass cytometric functional profiling of acute myeloid leukemia defines cell-cycle and immunophenotypic properties that correlate with known responses to therapy. Cancer Discov..

[126] Alexander G.M., Huang Y.Z., Soderblom E.J., He X.P., Moseley M.A., McNamara J.O. (2017). Vagal nerve stimulation modifies neuronal activity and the proteome of excitatory synapses of amygdala/piriform cortex. J. Neurochem..

[127] Nemes P., Knolhoff A.M., Rubakhin S.S., Sweedler J.V. (2011). Metabolic differentiation of neuronal phenotypes by single-cell capillary electrophoresis-electrospray ionization-mass spectrometry. Anal. Chem..

[128] Nemes P., Knolhoff A.M., Rubakhin S.S., Sweedler J.V. (2012). Single-cell metabolomics: Changes in the metabolome of freshly isolated and cultured neurons. ACS Chem. Neurosci..

[129] Onjiko R.M., Portero E.P., Moody S.A., Nemes P. (2017). In Situ Microprobe Single-Cell Capillary Electrophoresis Mass Spectrometry: Metabolic Reorganization in Single Differentiating Cells in the Live Vertebrate (Xenopus laevis) Embryo. Anal. Chem..

[130] Onjiko R.M., Moody S.A., Nemes P. (2015). Single-cell mass spectrometry reveals small molecules that affect cell fates in the 16-cell embryo. Proc. Natl. Acad. Sci. USA.

[131] Onjiko R.M., Morris S.E., Moody S.A., Nemes P. (2016). Single-cell mass spectrometry with multi-solvent extraction identifies metabolic differences between left and right blastomeres in the 8-cell frog (Xenopus) embryo. Analyst.

[132] Lombard-Banek C., Reddy S., Moody S.A., Nemes P. (2016). Label-free Quantification of Proteins in Single Embryonic Cells with Neural Fate in the Cleavage-Stage Frog (*Xenopus laevis*) Embryo using Capillary Electrophoresis Electrospray Ionization High-Resolution Mass Spectrometry (CE-ESI-HRMS). Mol. Cell. Proteom..

[133] Lombard-Banek C., Moody S.A., Nemes P. (2016). Single-Cell Mass Spectrometry for Discovery Proteomics: Quantifying Translational Cell Heterogeneity in the 16-Cell Frog (Xenopus) Embryo. Angew. Chem. Int. Ed..

[134] Hofstadler S.A., Swanek F.D., Gale D.C., Ewing A.G., Smith R.D. (1995). Capillary Electrophoresis-Electrospray Ionization Fourier Transform Ion Cyclotron Resonance Mass Spectrometry for Direct Analysis of Cellular Proteins. J. Neurosci. Methods.

[135] Mellors J.S., Jorabchi K., Smith L.M., Ramsey J.M. (2010). Integrated microfluidic device for automated single cell analysis using electrophoretic separation and electrospray ionization mass spectrometry. Anal. Chem..

[136] Valaskovic G.A., Kelleher N.L., McLafferty F.W. (1996). Attomole Protein Characterization by Capillary Electrophoresis-Mass Spectrometry. Science.

[137] Smith R.D., Shen Y., Tang K. (2004). Ultrasensitive and Quantitative Analyses from Combined Separations-Mass Spectrometry for the Characterization of Proteomes. Acc. Chem. Res..

[138] Cecala C., Sweedler J.V. (2013). Sampling techniques for single-cell electrophoresis. Analyst.

[139] Zhu Y., Zhao R., Piehowski P.D., Moore R.J., Lim S., Orphan V.J., Paša-Tolić L., Qian W.J., Smith R.D., Kelly R.T. (2018). Subnanogram proteomics: Impact of LC column selection, MS instrumentation and data analysis strategy on proteome coverage for trace samples. Int. J. Mass Spectrom..

[140] Zhang L., Khattar N., Kemenes I., Kemenes G., Zrinyi Z., Pirger Z., Vertes A. (2018). Subcellular Peptide Localization in Single Identified Neurons by Capillary Microsampling Mass Spectrometry. Sci. Rep..

[141] Zhang L., Vertes A. (2015). Energy Charge, Redox State, and Metabolite Turnover in Single Human Hepatocytes Revealed by Capillary Microsampling Mass Spectrometry. Anal. Chem..

[142] Zhang L., Foreman D.P., Grant P.A., Shrestha B., Moody S.A., Villiers F., Kwak J.M., Vertes A. (2014). In Situ metabolic analysis of single plant cells by capillary microsampling and electrospray ionization mass spectrometry with ion mobility separation. Analyst.

[143] Lee C.Y., Fan Y., Rubakhin S.S., Yoon S., Sweedler J.V. (2016). A neuron-in-capillary platform for facile collection and mass spectrometric characterization of a secreted neuropeptide. Sci. Rep..

[144] Budnik B., Levy E., Slavov N. (2017). Mass-spectrometry of single mammalian cells quantifies proteome heterogeneity during cell differentiation. bioRxiv.

[145] Wan H., Tang B., Liao X., Zeng Q., Zhang Z., Liao L. (2018). Analysis of neuronal phosphoproteome reveals PINK1 regulation of BAD function and cell death. Cell Death Differ..

[146] Distler U., Schmeisser M.J., Pelosi A., Reim D., Kuharev J., Weiczner R., Baumgart J., Boeckers T.M., Nitsch R., Vogt J. (2014). In-depth protein profiling of the postsynaptic density from mouse hippocampus using data-independent acquisition proteomics. Proteomics.

[147] Meier F., Geyer P.E., Virreira Winter S., Cox J., Mann M. (2018). BoxCar acquisition method enables single-shot proteomics at a depth of 10,000 proteins in 100 minutes. Nat. Methods.

[148] Ping L., Duong D.M., Yin L., Gearing M., Lah J.J., Levey A.I., Seyfried N.T. (2018). Data Descriptor: Global quantitative analysis of the human brain proteome in Alzheimer’s and Parkinson’s Disease. Nat. Publ. Gr..

[149] Carlyle B.C., Kitchen R.R., Zhang J., Wilson R., Lam T., Rozowsky J.S., Williams K.R., Sestan N., Gerstein M., Nairn A.C. (2018). Isoform level interpretation of high-throughput proteomic data enabled by deep integration with RNA-seq. J. Proteome Res.

[150] Menschaert G., Van Criekinge W., Notelaers T., Koch A., Crappé J., Gevaert K., Van Damme P. (2013). Deep proteome coverage based on ribosome profiling aids MS-based protein and peptide discovery and provides evidence of alternative translation products and near-cognate translation initiation events. Mol. Cell. Proteom..

[151] Dammer E.B., Duong D.M., Diner I., Gearing M., Feng Y., Lah J.J., Levey A.I., Seyfried N.T. (2013). Neuron Enriched Nuclear Proteome Isolated from Human Brain. J. Proteome Res.

[152] Zhu Y., Piehowski P.D., Zhao R., Chen J., Shen Y., Moore R.J., Shukla A.K., Petyuk V.A., Campbell-Thompson M., Mathews C.E. (2018). Nanodroplet processing platform for deep and quantitative proteome profiling of 10-100 mammalian cells. Nat. Commun..

[153] Tcherkezian J., Brittis P.A., Thomas F., Roux P.P., Flanagan J.G. (2010). Transmembrane Receptor DCC Associates with Protein Synthesis Machinery and Regulates Translation. Cell.

[154] Chen A.L., Kim E.W., Toh J.Y., Vashisht A.A., Rashoff A.Q., Van C., Huang A.S., Moon A.S., Bell H.N., Bentolila L.A. (2015). Novel components of the toxoplasma inner membrane complex revealed by BioID. MBio.

[155] Chen C.-L., Hu Y., Udeshi N.D., Lau T.Y., Wirtz-Peitz F., He L., Ting A.Y., Carr S.A., Perrimon N., Axelrod J.D. (2015). Proteomic mapping in live Drosophila tissues using an engineered ascorbate peroxidase. Proc. Natl. Acad. Sci. USA.

